# Integration of Body Sensor Networks and Vehicular *Ad-hoc* Networks for Traffic Safety

**DOI:** 10.3390/s16010107

**Published:** 2016-01-15

**Authors:** Angelica Reyes-Muñoz, Mari Carmen Domingo, Marco Antonio López-Trinidad, José Luis Delgado

**Affiliations:** 1 Computer Architecture Department, UPC BarcelonaTech, Esteve Terradas, Castelldefels 7-08860, Spain; 2 Electrical Engineering Department, UPC BarcelonaTech, Esteve Terradas, Castelldefels 7-08860, Spain; mari.carmen.domingo@upc.edu; 3 Laboratorio Nacional de Informática Avanzada (LANIA), Monterrey IT Cluster, Technological Research and Innovation Park (PIIT), Km. 10 Autopista al Aeropuerto Internacional Mariano Escobedo, Monterrey-Nuevo León 66629, México; mlopez@lania.mx (M.A.L.-T.); isc.jldelgado@gmail.com (J.L.D.)

**Keywords:** body sensor network, vehicular *ad hoc* networks, driver behavior

## Abstract

The emergence of Body Sensor Networks (BSNs) constitutes a new and fast growing trend for the development of daily routine applications. However, in the case of heterogeneous BSNs integration with Vehicular *ad hoc* Networks (VANETs) a large number of difficulties remain, that must be solved, especially when talking about the detection of human state factors that impair the driving of motor vehicles. The main contributions of this investigation are principally three: (1) an exhaustive review of the current mechanisms to detect four basic physiological behavior states (drowsy, drunk, driving under emotional state disorders and distracted driving) that may cause traffic accidents is presented; (2) A middleware architecture is proposed. This architecture can communicate with the car dashboard, emergency services, vehicles belonging to the VANET and road or street facilities. This architecture seeks on the one hand to improve the car driving experience of the driver and on the other hand to extend security mechanisms for the surrounding individuals; and (3) as a proof of concept, an Android real-time attention low level detection application that runs in a next-generation smartphone is developed. The application features mechanisms that allow one to measure the degree of attention of a driver on the base of her/his EEG signals, establish wireless communication links via various standard wireless means, GPRS, Bluetooth and WiFi and issue alarms of critical low driver attention levels.

## 1. Introduction

The behavior of the driver is essential for traffic safety. Fatigue, alcohol, on-road distractions and emotional state disorders deteriorate the driver’s performance, his/her assessment capability and may lead to loss of vehicular control and traffic accidents. Drowsiness is a significant contributing factor in traffic crashes. The American Automobile Association (AAA) estimates that 21% of deadly traffic accidents and 13% of crashes requiring hospitalization of car drivers or passengers are due to drowsy driving [1].

Drunk driving increases the likelihood of a road traffic crash and that death or serious injury will result [2]. Statistics show that in most high-income countries the proportion of fatally-injured drivers with excess alcohol is about 20%. In addition, in low-income countries drivers had consumed alcohol in 33% to 69% of crashes in which drivers were fatally injured and in 8% to 29% of crashes in which drivers were not fatally injured [3].

Distractions can also lead to impaired driving. The National Highway Traffic Safety Administration (NHTSA) estimates that 10% of the fatalities in 2014 were caused by a driver’s distraction and 3179 people died as a result in the United States [4].

The emotions of drivers (stress, frustration, *etc.*) may also endanger traffic safety. The inability to manage emotions has been identified as one of the major causes of accidents [5].

A Body Sensor Network is formed by multiple sensors placed at the human body to monitor the vital signs of a person [6]. The physiological states of the person are being sensed, sampled and processed by specific implant and body surface sensors. On the other hand, Vehicular *ad hoc* Networks (VANETs) [7] are mobile *ad-hoc* networks that enable the communication among nearby vehicles, and between vehicles and roadside equipment. In this work, an integrated BSN-VANET architecture is introduced. The proposed architecture focuses on how to avoid traffic accidents caused by an inadequate behavior of the driver.

This paper identifies how BSNs are useful to detect four basic behavior states (drowsy, drunk, driving under emotional state disorders and distracted driving) that may cause traffic accidents. If the driver’s state impairs driving, notification messages will be sent to the VANET to alert other vehicles and/or the emergency services. In addition, experiments in real driving conditions have been carried out to validate our proposal.

The structure of the paper is as follows: Section 2 discusses the state of art. Section 3 introduces our scenario. Section 4 explains in depth each one of the components of the proposed architecture. In Section 5, the monitoring station architecture is described. Section 6 compares the proposed architecture. Section 7 shows our experiments in a real life scenario. Section 8 discusses the performance of the proposed architecture. Finally, the main research challenges to be addressed and conclusions are summarized in Section 9 and Section 10, respectively.

## 2. State of the Art

Until now there is no agreement on how to measure and classify emotions. Some studies have been made such as a research area called Driving Psychology which is a behavioral engineering tool that operates in three separate but interacting behavioral areas: affective, cognitive, and sensorimotor [8].

We argue in this paper that some traffic accidents can be avoided thanks to a proper integration of BSNs and VANETs. The BSNs help us to detect if the emotional state of the driver could produce driving impairment. Our research focuses on four behaviour states that cause serious driving impairments: drowsy driver, drunk driver, driver with emotional disorders and distracted driver.

### 2.1. Drowsy Driving Detection

The typical visual clues when a person feels drowsy are frequent yawning, frequent eye-blinking, pupil movement (gaze), head movement and facial expression. Several systems have been developed for the detection of drowsiness driving using physiological parameters such as eye movement measures (electrooculogram, EOG), heart activity (electrocardiogram, ECG), skin electrical potential (electromyogram, EMG) and brain activity (electroencephalogram, EEG). In [9], a real-time wireless EEG-based computer interface system for drowsiness detection is proposed. It consists of a wireless physiological signal-acquisition module, which collects EEG signals with an electrode, amplifies, filters, pre-processes and sends them to the embedded signal-processing module using wireless communication. The signal-processing module detects real-time drowsiness. The wireless physiological signal-acquisition module can be embedded into a headband as a wearable device being part of the BSN. A warning tone is triggered to warn the driver when a drowsy state occurs. In [10], a driver fatigue recognition model based on the dynamic Bayesian network is proposed. Multiple features (contextual such as sleeping quality or work environment, contact physiological such as ECG or EEG and contactless physiological such as eye movements) were used; it is shown that the EEG and ECG signals contribute significantly to fatigue detection. The first-order Hidden Markov Model (HMM) has been employed to compute the dynamics of a Bayesian network. Simulation-based experiments were performed to validate the proposed model. In [11], a driver fatigue and drowsiness monitoring system based on an ECG sensor attached to the steering wheel is proposed. The heart rate variability of the ECG signals measured from the driver palms is analyzed; it enables the non-intrusive detection of drowsiness and fatigue condition in the driver. In [12], eyelid parameters are extracted from the EOG data collected in a simulated driving experiment; Support Vector Machine (SVM) is the technique used to detect successfully the drowsiness of the drivers. In [13], a non-invasive driver assistance system is introduced. It is able to detect ECG/EEG signals through cloth or hair without direct skin-contact. Eye blinking can also be detected at a distance of 10 cm. The experiments carried out using a driving simulator show that the blinking duration as well as the heart rate variability (HRV) are useful physiological measures to distinguish between alertness and drowsiness. In [14] authors propose a system that indicates the driver’s fatigue level using electrocardiography, heart rate variability, blood pressure and photoplethymosgraphy signals collected from an indoor driving simulation. If their fuzzy Bayesian network probabilities are between the values of 0.30 and 0.54, this means that the subject is fully awake, if the probability interval increases to 0.60 the driver is starting to get drowsy and between 0.60 and 0.75 it is considered that the driver is in the partial-sleep condition. In [15], the authors state that the reliability and accuracy of driver drowsiness detection by using physiological signals is very high compared to other methods such as vehicle-based measures and behavioral measures.

There are some techniques that measure physical changes during fatigue such as computer vision. A digital camera is used in [16] to compute the driver’s eye state and eye blinking frequency, head movement and sagging body posture. In [17], a driver monitoring and event detection system based on 3D information from a range camera is presented. A Kinect sensor integrated in a commercial vehicle allows head pose estimation, which enables the detection of driver’s drowsiness or inattention.

### 2.2. Drunk Driving Detection

Alcohol affects eye-to-hand reaction times and the brain control of eye movements is also highly vulnerable to alcohol. Blood alcohol concentrations (0.03% to 0.05%) interfere with voluntary eye movements and impair the eye’s ability to rapidly track a moving target.

Blood alcohol concentration can be measured using interstitial fluid alcohol level sensors belonging to a BSN. The authors in [18] have developed an armband hosting an interstitial fluid based alcohol sensor that enables real-time collection of blood-alcohol level. The sensor responds to a drinking event and the data is highly correlated with the blood alcohol concentration. The data is made available to an attached mote which processes it, stores it locally, and transmits it to a remote server via a mobile phone. In [19] the authors propose to sense the level of driver’s alcohol using the PAS 32U alcohol sensor. If the sensor detects 10% of alcohol in the breath of the driver, then the speed of the vehicle is cut down to 80 km/h automatically. Between 20% and 30% of alcohol content the speed of the vehicle is maintained at 60 km/h. When an alcohol content of more than 30% is detected, a caution message is displayed on the LCD screen. If the driver ignores the message and does not stop the vehicle in the stipulated time, the system stops the vehicle automatically and sends an SMS alert to law and enforcement authority with the GPS location. In [20], the authors define a sensing phase to get information about alcohol levels in the driver’s blood, the direction of driver’s eyes, *etc.* The reasoning phase is responsible for extracting the situation of the driver and calculating corrective actions for other vehicles on the road. Finally the application phase disseminates warning messages for other vehicles on the road and warns the driver to prevent the occurrence of accidents.

In [21] the system authenticates the driver by combining the function of alcohol detection and a face identification system based on image recognition. It offers to eradicate the fraudulent conduct of drunk driver and driver changing. A sensor reads the alcohol concentration of the air around the driver and determines whether it is less than the original standard. If it is higher, the ignition circuit of the engine is cut off by the On Board Unit (OBU) to hinder driving. It will only be possible to start the car if the alcohol concentration is reduced.

In [22] the authors propose a seat incorporating a sensor that can be retrofitted into an automobile seat for detection of impairment of a driver who has consumed alcohol. Body-trunk plethysmogram and respiration are detected from the back of the driver using the body sensor. The extracted signal was defined as a pulse wave that allows a quick detection of alcohol-impaired driving. In [23] alcohol content in the driver’s body is detected by means of an infrared breath analyser placed in the steering wheel. The alcohol level of the driver is continuously monitored and calibrated on a scale. When it exceeds a particular limit the fuel supply is cut-off.

In [24] there is an overview that focuses on in-vehicle capability to detect impairment before a vehicle can be operated. They mention a biometric fingerprint device capable of detecting alcohol levels (this device is placed on the steering wheel to detect alcohol through the pores of the skin); alcohol sniffers to measure the driver's’ breath (placed on the driver’s seat, behind the driver’s shoulder); transdermal sensors that measure blood alcohol concentration based on how much alcohol is present in perspiration (this device is worn around the ankle).

### 2.3. Detection for Driving under Emotional State Disorders

Emotions are mental and physiological states associated with a wide variety of feelings, thoughts, and behaviors. Emotional state disorders impair driving. Some works have evaluated with BSNs the emotional state of the drivers in simulated environments. In [25], a methodology and a wearable system for the evaluation of the emotional states of car-racing drivers in simulated racing conditions have been introduced. The emotional states high or low stress, euphoria and disappointment have been determined by classifying features extracted from the facial electromyogram (EMG), electrocardiogram (ECG), respiration, and electrodermal activity (EDA) sensors*.* Support vector machines (SVMs) and Adaptive Neuro-Fuzzy Inference System (ANFIS) have been used for the classification of the emotions with overall classification rates of 79.3% and 76.7%, respectively. In [26], a real time, wearable system for remote monitoring of the affective condition of car racing drivers has been presented. The emotional classes identified are high stress, low stress, dysphoria and euphoria. It has been validated with 10 subjects in simulated racing conditions with a high classification. In [27], the impact of drivers’ emotions on driving performance has been investigated with fifteen participants in two driving simulation experiments. The results indicate the driver’s task performance varies according to different emotional states. In [28], the electroencephalogram (EEG) signals have been analyzed in a car simulation environment with and without billboards. It has been observed that the registration of billboards generates a specific brain signal; consequently, the blinking rate and the driver’s stress level are increased. In [29], the authors evaluate the driver’s stress level analysing the autonomic nervous system changes using sensors to measure heart rate variability, respiration activity and electrodermal response along with mechanical information such as that coming from steering wheel angle corrections, velocity changes and time responses. Fifteen subjects have performed a driving simulation experiment consisting of a first session keeping a constant speed in a motorway and two additional sessions in the same environment with incremental stress load. In [30], the authors propose a monitoring system that alerts people before they become sick and prevents them from having motion sickness symptoms while driving a car. The authors use EEG features that have been extracted online from five motion sickness-related brain areas. A virtual reality-based dynamic driving simulator has been constructed to perform experiments. The results show that motion sickness has been successfully detected in more than 80% of the subjects. The experiments of [25,26,27,28,29,30] have been taken in simulated conditions. However, emotions do not always correlate with specific driving events [25] and subjects experience different emotions to driving events on simulation environments from those in real conditions.

Other empirical studies have also analyzed with BSNs the driver’s emotions in a real life scenario. In [31], the authors show that driver’s physiological signals were measured with acceptable quality of analysis without interrupting driving. Authors defined two categories of stresses using nonintrusive sensors (electrocardiogram, photoplethysmogram, galvanic skin response and respiration) on the steering wheel, driver’s seat, and seat belt. In [32], physiological sensing has been applied to determine the driver’s stress level during real-world driving tasks. ECG, EMG, EDA and respiration have been recorded during a set route, which included periods of rest, highway and city driving. A driver questionnaire and a score derived from observable events and actions coded from a videotape taken during the drives have been used to assess the stress levels. The results show that three stress levels (low, medium, and high) could be recognized with an overall accuracy of 97.4% using 5 min intervals of data and that heart rate and skin conductivity metrics provide the highest overall correlations with continuous driver stress levels. The authors in [33] describe how to use combinational fusion to select features and fuse physiological sensor data to accurately detect stress level during driving. They use the data acquired by Healey and Picard’s experiments [31] for feature extraction. In [34], a real-time methodology for the assessment of drivers’ stress has been introduced. The detection has been based on physiological signals (ECG, EDA and respiration) as well as driving history extracted using GPS and the vehicle’s CAN-bus data. This information has been incorporated in a Bayesian network (BN) to estimate the stress probability. Stress events can be detected through monitoring of the driver’s physiological reactions to driving events; the results in real driving conditions show an accuracy in stress event detection of 82%. In [35], the driver’s affective state has also been monitored using physiological signals (EDA and photoplethysmography (PPG)) during on-road driving experiments. Neural networks have been used to predict the driver’s stress levels with an average precision of 89.93%. In [36], the driver’s frustration in a real world scenario has been inferred by a BN given the driving environment (e.g., traffic density or road obstructions), speech recognition errors during the interaction with an Automatic Speech Recognition (ASR) system to retrieve and play music, EDA, face expressions and pedal actuation. The system shows a true positive rate in frustration detection of 80% and a false positive rate of 9%. In [37], the authors present a methodology for stress detection in realistic driving conditions. Their signal acquisition process considers an ECG sensor which is placed on the subject’s chest, electrodermal activity sensors are attached on the subject’s middle, index fingers on the right hand, and a respiration sensor is placed around the subject‘s thorax. The most significant indicators have been selected and a dataset has been collected during real-world driving. Experiments have been carried out in a simulated environment to study how the estimated changes of driver’s state affect driving performance. In [38] heart rate, respiration rate and hand pressure is used to measure stress. Authors analyze the loss of peripheral vision and explain that the car could compensate for this by auto-adjusting the field of view of headlights; also authors propose to change the color of the dashboard based on physiological changes (green and red colours could indicate a more relaxed or stressed driver, respectively).

### 2.4. Distracted Driving Detection

A distraction is anything that diverts the driver’s attention from the primary task of navigating the vehicle and responding to critical events. In [39] motion sensors are used to detect distractions. The abnormal movements of the head or leg are an indication of driver distractions (such as an abrupt brake or a sharp turn), which can be detected using accelerometers and gyroscopes. The current vehicle signals vehicle speed, brake, acceleration and steering angle are extracted from the Controller Area Network (CAN) bus and are combined with motion sensor data to identify the distractions. This way, a high accuracy of distraction detection of over 90% is achieved. In [40], a driver distraction monitoring system based on dual compass motion sensing is proposed; it is able to recognize driver’s head pose by removing the vehicle motion component, which also affects head pose recognition. The driver’s distraction index was computed and compared to a threshold to warn the driver if necessary. In [41], a non-intrusive distracted driving warning system based on motion capture technology is introduced. It is composed of: (i) the Microsoft Kinect motion sensing hardware for tracking head and skeletal movements and (ii) a custom software application for identifying four distractions and outputting audio alerts. Four different distractions can be detected: (a) reaching for a moving object; (b) talking on a cell phone; (c) personal hygiene; and (d) looking at an external object. The system is able to successfully identify these distractions in a driving simulator with accuracy of (a) 100%; (b) 33%; (c) 50%; and (d) 66%. Drivers are alerted of their distractions with audio signals.

In [42], the distraction level of a driver performing secondary tasks (talking in the phone or to a passenger, operating a navigation system or changing radio stations) is analyzed. The driver’s distractions are detected in real-world scenarios by the CAN bus, a frontal camera facing the driver and a microphone. The CAN bus provides relevant car data such as brake value, acceleration, vehicle speed, steering wheel angle, brakes and gas pedal pressures. A video camera captures the facial expressions and head orientation of the driver and a microphone array provides audio. Another camera is placed facing the road. Separate binary classifiers are trained to recognize whether the driver is performing some secondary tasks, achieving an average accuracy of 77.2%. A joint multi-class classifier is trained and achieves an accuracy of 40.8%. Afterwards, a regression model to predict the level of distraction of the driver is built; it highly correlates with the perceived driver distraction scores from subjective evaluations. In [43], regression models were used to predict the perceived distraction level of the drivers. The independent variables are features extracted from the CAN-bus signal, a microphone array and two video cameras facing the road and the driver. Four different distraction modes were identified using the proposed system. In [44], algorithms and experimental results to detect distraction by using in-vehicle signals are introduced. The variance of steering angle and average steering speed were selected as most distinguishing signals between normal and distracted driving. Two kinds of machine learning schemes (unsupervised learning and supervised learning together) are used to classify normal and distracted driving features in real driving situations.

In [45], a distributed camera framework for continuously monitoring the driver’s head dynamics is proposed. The system has been evaluated during on-road driving with spatial head turns. The results show that the proposed system was reliable over 90% of the time in comparison with the single-perspective approach (reliability less than 72% of the time). In [46], a distributed camera framework for head movement analysis is introduced. Two approaches for facial feature tracking to compute the head pose and different configurations of multi-camera systems have been analyzed. The head movement could be tracked by the best system over 96% of the time. Evaluations were performed in real-world scenarios to capture spatially large head turns (away from the driving direction) during different maneuvers (e.g., merge and lane change) on urban streets and freeways. The system in [47] requires monocular video for real-time driver head pose estimation, since head pose indicates the current focus of attention of the driver. New algorithms for automotive head pose estimation and tracking have been introduced. A new detection method based on video camera is proposed in [48]. The new algorithm includes driver’s face location based on gray variance, and segmentation of separate areas of facial organs using the projection curve pole position. It is able to distinguish between distraction and fatigue states according to the driver’s head rotation angle. The results show a good average real-time performance (32 ms) and a high accuracy rate.

In [49] texting during driving is detected with the aid of smartphones. Motion sensors integrated in the smartphone are able to detect who the driver is (sitting on the front seat and right side of the car) recognizing micro-movements. A naïve Bayesian classifier has been chosen; it achieves an accuracy of 87.18% and a precision of 96.67%.

The online monitoring system in [50] detects early driver distractions using brain activity measured by EEG signals. The proposed method predicts the start and end of a distraction period. 24 subjects were asked to drive from an initial position to a destination using a city map in a simulated driving environment. The experimental results showed that the algorithm is able to predict the start and end of distraction periods with relatively high accuracy (81% and 70%, respectively). In [51], the driver’s EEG signal is analyzed to detect cognitive distractions. A new EEG analysis method based on Singular Value Decomposition (SVD) is introduced. The proposed method has been analyzed in a highway-driving simulated environment with a high accurate detection of the driver’s cognitive state.

Table 1 summarizes the various different body sensors used to detect drowsy driving, drunk driving, driving under emotional state disorders and distracted driving.

**Table 1:** Sensors to detect four behavior states that cause driving impairments.

State	Sensor	Data Rate	Bandwidth
Drowsy driver	ECG	288 Kbps	100–1000 Hz
	EEG	43.2 Kbps	0–150 Hz
	EOG	1.2 Kbps	0.05–35 Hz
	EMG	320 Kbps	0–10,000 Hz
Drunk driver	Interstitial fluid	–	–
	Plethysmogram	2400 bps	0.5–4 Hz
	Respiration	800 bps	0.1–10 Hz
Driver with emotional disorders	Respiration	800 bps	0.1–10 Hz
	EDA	1.2 Kbps	0–35 Hz
	Glucose	1600 bps	0–50 Hz
	Facial EMG	320 Kbps	0–10,000 Hz
	ECG	288 Kbps	100–1000 Hz
Distracted driver	EEG	43.2 Kbps	0–150 Hz
	EOG	1.2 Kbps	0.05–35 Hz
	Accelerometers, gyroscopes	35 Kbps	0–500 Hz

## 3. Proposed Scenario

**Figure 1:**
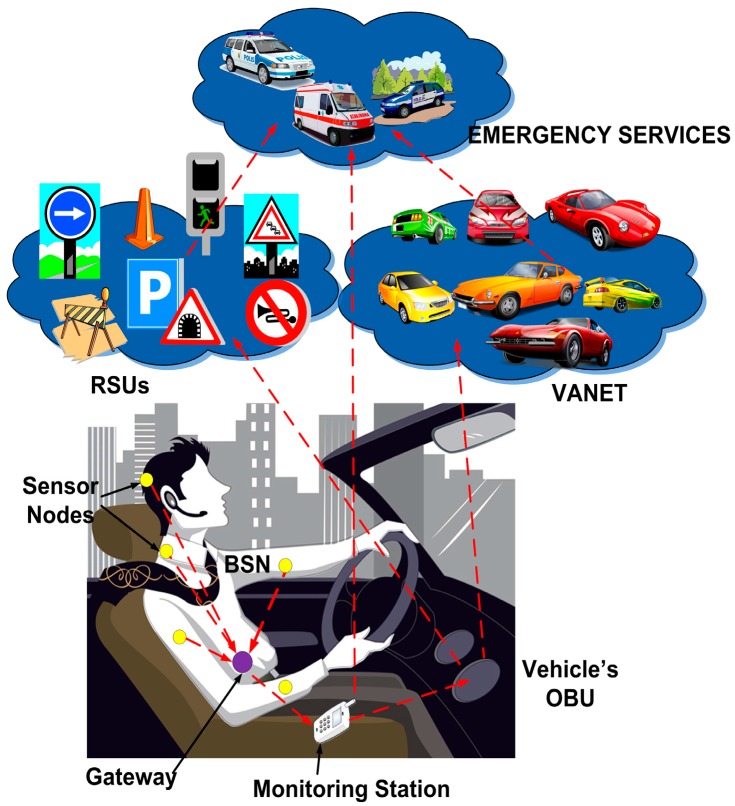
Proposed scenario.

We propose a scenario (see Figure 1) where the behavior of the driver is monitored to avoid traffic accidents. Vital signs (body temperature, heart rate, blood pressure, respiratory rate, *etc.*) of the driver are measured and sent to a gateway located on the body surface, which forwards this data to the monitoring station. The monitoring station is a wireless device such as cell phone, tablet PC, smartphone, *etc.*, which is able to connect to the vehicle’s OBU. The OBU is a network device that communicates with other vehicles (VANET) and with fixed infrastructure.

In this scenario, the vital signs sent by the BSN are analyzed in the monitoring station to determine the driver’s state. Four common causes of traffic accidents due to an irresponsible behavior of the driver have been selected: drowsy, drunk, driving under emotional state disorders, and distracted driving. Since these ways of driving constitute a danger to road users, the surrounding vehicles and pedestrians should be advised for safety reasons.

If the behavior of the driver is inadequate, notification messages are sent from the monitoring station to the vehicle’s OBU, which forwards them to the VANET and/or the Road Side Units (RSUs). Our architecture contemplates that the notification messages in the VANET use the current VANET protocols. Notification messages between the OBU and the emergency services or the RSUs use the available communications platforms such as 4G, WiMAX, *etc.* Pedestrians should also be warned through these notification messages, which are forwarded to their own monitoring stations (e.g., smart phones) using Vehicle to Pedestrian (V2P) or Infrastructure to Pedestrian (I2P) communications. Next, we introduce the proposed system architecture.

## 4. Modular Integration of BSN and VANET

**Figure 2:**
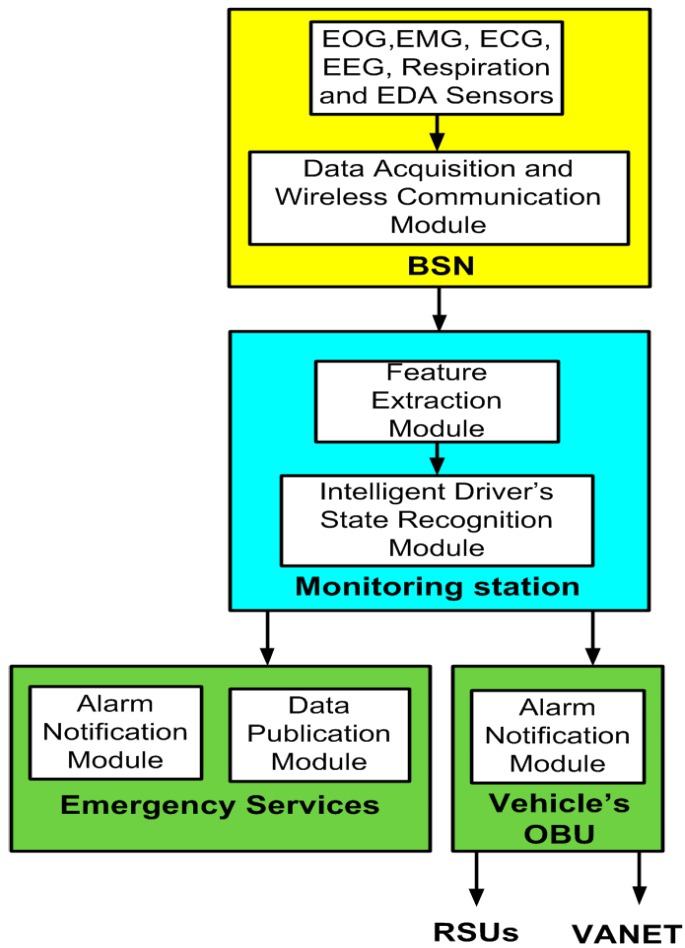
Integrated BSN and VANET architecture.

The proposed integrated BSN and VANET architecture is shown in Figure 2. It consists of: (1) the BSN; (2) the monitoring station; (3) the vehicle’s OBU and (4) the emergency services. Next, we describe its components and functionalities in detail.

### 4.1. Body Sensor Network

The following sensors have been used to monitor the driver state at a specific moment: electrocardiogram (ECG), electroencephalogram (EEG), electrooculogram (EOG), facial electromyogram (EMG), interstitial fluid, plethysmogram, respiration, electrodermal activity (EDA), glucose and motion sensors (accelerometers and gyroscopes).

ECG is a graphic record of the heart’s electrical activity. The ECG sensors are located on the thorax of the driver. The EEG sensor of the head measures the electrical activity within the brain. The EOG is the resulting signal when the resting potential of the retina is measured. It records eye movements. The EOG sensors are placed around the eye. The EMG sensor measures the muscle activity or the frequency of tension of a certain muscle. A balaclava contains the EMG sensors.

The interstitial fluid based alcohol sensor enables real-time collection of blood-alcohol level. Interstitial fluid is harvested via micro pores and assayed in real time for blood alcohol content. An arm band hosts this sensor.The plethysmogram shows fluctuations in pulse or heartbeat frequency to judge whether a subject has been drinking alcohol. The plethysmogram is detected from the back of the driver.

Respiration is an indicator of how deep and fast a person is breathing. Respiration sensors are located on the thorax of the driver. EDA refers to the skin conductance activity. The EDA sensor could be placed inside a driver’s glove or on the neck. The glucose sensor placed under the skin measures the amount of glucose circulating in the blood.

An accelerometer is used to recognize and monitor body posture. A gyroscope is used for measuring or maintaining orientation, based on the principle of conservation of angular momentum. Gyroscopes together with accelerometers monitor the physical movements. Motion sensors can be placed on the leg and the head.

The acquired data is transmitted via a wireless communication module. Ultra-wideband or IEEE 802.15.6 [52] is used for wireless transmission between the sensors and the gateway. Bluetooth or ZigBee can be used to forward the physiological data from the gateway to the monitoring station [53]. Different MAC protocols have also been developed for the communication in wireless body sensor networks [54,55] and cognitive radio wireless body sensor networks [56].

### 4.2. Monitoring Station

The acquired data from the BSN is processed in the monitoring station because the BSN gateway is restricted by its low capabilities and limited battery capacity. The monitoring station is the core component of the system. It is divided into two major modules, namely: the feature extraction module and the intelligent driver’s state recognition module. The first module extracts features from the selected bio signals. These features are used by the intelligent driver’s state recognition module to determine if the driver has one of the four predefined subject’s states (drowsy, drunk, emotional state disorder or distracted driver). The state is being monitored during the driving task. More details about monitoring station components are explained in Section 5.

### 4.3. Vehicle’s OBU

The OBU has an alarm notification module. If impaired driving is detected due to an inadequate behavior of the driver, then alarm notification messages are sent from the monitoring station towards the OBU, which forwards them to the VANET and/or the RSUs.

### 4.4. Emergency Services

We distinguish between the alarm notification and the data publication modules. The emergency services can also be notified of an inadequate driver’s state if it is required to assist the driver. The collected information of body sensors is also sent from the monitoring station to the emergency services for police control and/or medical reasons. OpenXDF has been selected as an open XML-based standard for the digital storage and exchange of time-series physiological signals and metadata between the monitoring station and the vital sign monitoring server of the emergency services. The resulting XML documents are sent to this server to be published and accessed by authorized users. Next, we describe the components of the monitoring station architecture.

## 5. Monitoring Station Components

**Figure 3:**
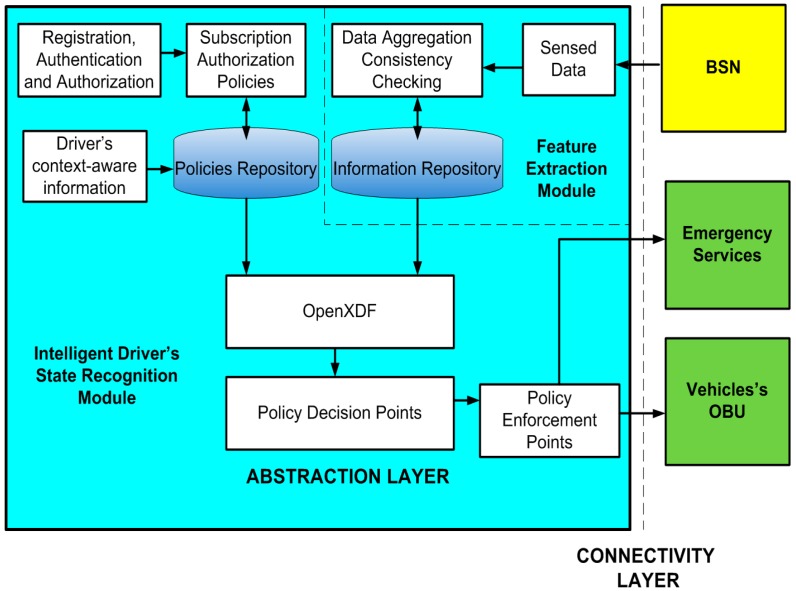
Monitoring Station Architecture.

The monitoring station architecture is shown in Figure 3. The architecture consists of connectivity and an abstraction layer. The connectivity layer has an interface for the BSN, another for the emergency services and one last for the OBU. In the abstraction layer, body sensor data should be processed, formatted and sent to the emergency services in case of impaired driving due to the driver’s state.

The registration, authentication and authorization module controls the security and privacy between the monitoring station and the OBU and between the monitoring station and the emergency services. Afterwards, subscription authorization policies are defined for the monitoring station interaction with the OBU and between the monitoring station and the emergency services.

We incorporate policy rules to prioritize information previously defined and stored in the policies repository. These rules are related to the appropriate laws of the driver’s country (e.g., the legal blood alcohol concentration limit for driving) and the steps to take in case of impaired driving due to an inadequate behavior of the driver (see Section 5.1). Also, there is a driver’s context aware information module in charge of feeding the policy repository with personal information such as: age, driving experience, *etc*. The purpose of all these rules is to help in the decision that determines if a person has a specific state that produces driving impairment.

The acquired data from the BSN is processed in the monitoring station where data aggregation and consistency checking techniques are applied. Afterwards, this data is stored in the information repository. Next, it is analyzed by the policy decision points. The information repository provides information to the policy decision module using OpenXDF.

The policy decision points are the architecture elements where all decisions are taken according to the established policies from the policy repository and the body sensor data which is stored in the information repository. The decision points select the adequate policy that should be applied in the enforcement points. The information repository uses OpenXDF for the digital storage and exchange of time-series physiological signals and metadata.

The enforcement points are involved in the execution of the policies. This module receives the action that should be applied in the OBU, as well as the transmission of notification messages to the OBU and to the emergency services. It should also send the sensor data to the vital sign monitoring server of the emergency services, which will be published based on the publication policies defined at the monitoring station.

### 5.1. Enforcement Points According to the Driver’s State

Once our architecture has detected impaired driving, we propose to have a variety of enforcement points and also linkages between multiple emergency services, for example having accessible the Driver’s context-aware information (see Figure 3) such as: driver identity confirmed through data and digital photo, contact information, conviction history, license status, medical problems, age, driving experience, *etc.* In [57] there is a proposal that law enforcement agencies may use the driver history to identify violators who have been encountered driving under impaired conditions.

**Figure 4:**
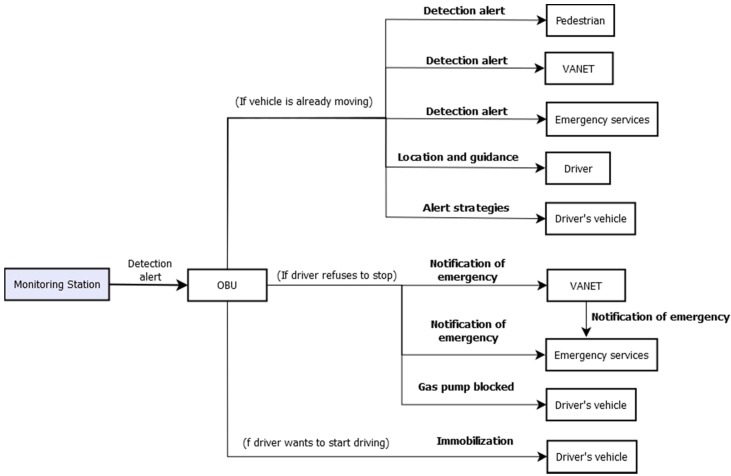
Enforcement points.

Roadside information would be also useful as context-aware data, for example: traffic density and weather conditions (this process shall manage data directly from collection equipment located at the roadside.). Figure 4 summarises the enforcement points in a general way for the four behavior states.

Next subsections detail the enforcement points when a detection alert (for drowsy driving, drunk driving, driving under emotional state disorders and distracted driving) is sent to the OBU.

#### 5.1.1. Enforcement Points for Drowsy Driving

The statistics from Section 1 demonstrate that drowsiness is a significant contributing factor in traffic accidents. Therefore, the driver should be notified when a drowsy state occurs so that he/she stops. Experts recommend drowsy drivers [58] to either pull over for a twenty-minute nap or let someone alert drive instead. It has been demonstrated that a short nap has the greatest effect on the driver’s performance and the effects last for several hours [59].

In addition, a drowsy detection alert is sent from the OBU to warn the other drivers (VANET) and pedestrians. This way, they could increase the security distance with respect to this vehicle and react more quickly in case of accident danger.

It is necessary to identify safe places for a nap [60]. For this purpose, Global Positioning System (GPS) applications for VANETs can be used [61]; they provide enhanced route guidance and navigation. Once the drowsy driver’s state is detected, the OBU enforces the GPS to locate the next road exit or service station. Information concerning hotels and accommodations could also be offered. The emergency lights are automatically switched on by the OBU.

In addition, the driver’s alert can be kept in his/her way towards the next service/road exit using specific alert strategies: opening the window, turning on the air conditioning, or playing loud music. These strategies are only emergency transient measures that give the driver some time to pull off the road [62]; however, its effectiveness to improve alertness when sleepy has not been demonstrated [58].

In case the driver refuses to stop, a notification message is automatically sent towards police cars using the VANET and/or towards the nearest police station. In the meantime, the fuel is cut off to prevent him/her from driving. If the driver decides to start again the vehicle and the BSN detects he/she is still drowsy, a notification message is sent to the OBU, which prevents driving by immobilizing the vehicle's motor.

#### 5.1.2. Enforcement Points for Drunk Driving

The statistics from Section 1 demonstrate that alcohol is a significant contributing factor in traffic accidents. Some factors that affect the absorption of alcohol include: person's weight, age, individual biomedical/chemical make-up, rate of metabolism and food in the body. This information is managed by the driver’s context aware information module (see Figure 3).

If a driver wants to start driving, but the BSN detects a higher alcohol concentration than the allowed concentration by law (in each country is different) then the vehicle will not start. The ignition will be locked out for some period of time. This method can ensure that the driver is sober prior to operating a motor vehicle [63]. Authors in [64] said that alcohol interlocks are associated with reductions in drunk driving by an average of 64%. In [65] showed that alcohol interlocks reduced the risk of committing an alcohol traffic violation within the first year by 65% while the device was installed.

Date, time, driver’s data and his/her ethanol concentration are sent to the emergency services. After some period of time the vital signs of the driver are sensed again. If the vehicle is already moving and the BSN detects an alcohol concentration that impairs driving, then the OBU of the vehicle should notify the driver about his or her own alcohol concentration and the GPS indicates the place to immediately stop. If the driver does not obey, the 3G system notifies the police indicating the GPS position of the vehicle. In the meantime, visual and auditory annoying warnings are signaled to the driver [66]. Another measure is to cut off the fuel [23] (the vehicle slows down, it does not stop abruptly) to prevent him/her from driving. Also, the OBU will send an alert message to the other vehicles in their geographical range using the VANET to increase the security distance from the drunk driver. This way, the rest of drivers have more time to react in case of a dangerous situation. Alert messages will be sent to pedestrians as well.

#### 5.1.3. Enforcement Points for Emotional State Disorders

The affective state recognition of drivers is essential for traffic safety. Driving under unacceptable levels of accumulated stress deteriorates vehicle control. During driving, drivers are affected by [67] events (sequential maneuvers such as stopping for the light, putting on the brakes, *etc.*) and sudden incidents like unanticipated pedestrian crossing, abrupt lane change by another vehicle, *etc.* Some stressors for urban bus drivers [68] include increased road traffic, violent passengers, and increasingly tight running schedules from commercial pressure.

The statistics from Section 1 demonstrate that emotional state disorders are a significant contributing factor in traffic accidents. For this reason it is required to monitor the driver’s affective state continuously, detecting on-road stress-trends and cumulatively accounting for increasing stress level [69]. Therefore, if the driver wants to start driving but the BSN detects impairment due to an emotional disorder, some countermeasures are taken to reduce anger and stress [70]: asking questions to the driver, offering breathing exercises, playing music, *etc.* Other strategies that can help the driver to better manage his/her stress are lowering the volume of music or listening to a more relaxing audio, temperature adaptation, corrective headlight [38] (see Section 2.3), reflective dashboard (see Section 2.3), *etc.* If the driver is still emotionally impaired, the vehicle will not start. If the vehicle is already moving and the BSN detects an abnormal emotional behavior, then the GPS system of the vehicle indicates the closest possible place to stop.

If the driver does not obey, the 3G system will notify it to the emergency services indicating the GPS position of the vehicle. Also, the OBU will send an alert message to the other vehicles in their geographical range using the VANET to help them to decide how to be as far away as possible of the indicated vehicle. This way, the rest of drivers have more time to react in case of a dangerous situation. Alert messages will be sent to pedestrians as well.

#### 5.1.4. Enforcement Points for Distracted Driving

The causes of distraction can be broadly classified into visual, cognitive, biomechanical and auditory [71]. On the other hand, researchers have found out that the distraction ‘latency’ lasts an average of 27 s [72], which means that, even after drivers put down the phone or stop fiddling with the navigation system, drivers aren’t fully engaged with the driving task. The statistics from Section 1 demonstrate that distractions are a significant contributing factor in traffic accidents. Teen drivers were distracted almost a quarter of the time they were behind the wheel [73]. 7% of the identified distractions were related to electronic devices such as texting, e-mails, and downloading music [73]. Other distractions included adjusting controls, eating or drinking, personal hygiene, reading, turning around, reaching for an object, and communicating with someone outside the vehicle. Using a cellphone while driving can also pose serious risks for traffic safety [74]. Therefore, the driver should be notified when he/she is distracted. If the glance-monitoring system detects that the driver is not looking ahead in some risky situations (e.g., when the radar detects a potential crash), the driver will receive a voice warning [75].

Other enforcement points include the use of GPS to locate and advertise the next road exit and voice alert suggestions of rest areas that offer services such as Internet access, which could encourage drivers to defer engagement in secondary tasks until they arrive at the rest area [75].

After three times advertising without any change of the behavior of the driver, he/she should be forced to abandon the road and take a break of a minimal time period. In case the driver refuses to stop, a notification message via the VANET will be automatically sent towards police cars and/or towards the nearest police station. In the meantime, the fuel will be cut off to prevent him/her from driving. In addition, once driver distractions are detected by the BSN, the system should notify it to the other drivers using the VANET. This way, the other drivers are warned and can slow down their vehicles or change lane to maintain a proper safety distance to prevent a possible accident.

## 6. Architecture Comparison

In Table 2, the main components of the proposed architecture are listed and then as a sort of comparison, the literature projects characteristics are crossed against our project listed features.

**Table 2:** Comparison of platform features offered by the current literature developments.

Platform	BSN	Behavior State	Monitoring Station	OBU	Emergency Services	Communication Interface	VANET
[9]	EEG	Drowsiness	No	No	No	Bluetooth	No
[10]	ECG,EEG	Fatigue	No	No	No	No	No
[11]	ECG	Fatigue, Drowsiness	No	No	No	No	No
[12]	EOG	Drowsiness	No	No	No	No	No
[13]	ECG,EEG	Alertness, Drowsiness	No	No	No	No	No
[14]	ECG, heart rate variability, blood pressure, photoplethysmosgram	Drowsiness	No	No	No	No	No
[15]	ECG,EMG,EOG, EEG	Drowsiness	No	No	No	No	No
[16]	Camera	Fatigue	No	No	No	No	No
[17]	Kinect System	Drowsiness	No	No	No	No	No
[18]	Interstitial fluid	Drunkness	Yes	No	Yes	TDMA 2.4Ghz. RF radio, Bluetooth	No
[19]	Fuel cells, breath analyser	Drunkness	No	No	Yes	No	No
[21]	Infrared breath analyser, camera	Drunkness	No	Yes	Yes	No	No
[22]	Back-pack sensor, body-trunk plethymogram and respiration	Drunkness	No	No	No	No	No
[23]	Infrared breath analyser	Drunkness	Yes	No	Yes	No	No
[25]	Facial electromyograms, ECG, respiration, and electro dermal activity	Emotional states	Yes	No	Yes	No	No
[26]	Facial expressions	high stress, low stress	No	No	No	No	No
[28]	EEG	Stress	No	No	No	No	No
[29]	Heart rate variability, respiration, electro dermal activity	Stress	No	Yes	No		No
[30]	EEG	Motion sickness	No	No	No	No	No
[31]	ECG, photoplethysmosgram, galvanic skin response, respiration	Stress	Yes	No	No	Bluetooth	No
[32]	ECG, electromyogram, skin conductance, respiration	Stress	No	No	No	No	No
[33]	EMG, skin conductance, respiration, heart rate	Stressfulness	No	No	No	No	No
[34]	ECG, electro dermal activity, respiration, driving behavior	Stress	No	Yes	No	No	No
[35]	EDA, photoplethysmosgram	Stress	No	No	No	No	No
[36]	Speech recognition	Frustration	No	Yes	No	No	No
[37]	ECG, electro dermal activity, respiration, face video recording,	Stress	No	No	No	No	No
[38]	Heart rate variability, electro dermal activity, respiration rate, voice, hand pressure	Stress	No	No	No	No	No
[39]	Accelerometer, gyroscope, driver leg, head movements	Distraction	No	Yes	No	No	No
[40]	Head posture		No	No	No	No	No
[41]	Kinect system	Distraction	Yes	No	Yes	No	No
[42]	Video camera, microphone array	Attention	No	No	No	No	No
[45]	Camera array	Distraction	No	No	No	No	No
[46]	Camera array	Distraction	No	No	Yes	No	No
[47]	Camera array	Awareness	No	No	No	No	No
[48]	Camera	Fatigue, Distraction	No	No	No	No	No
[49]	Smartphone accelerometer, texting	Texting while driving	No	No	No	No	No
[50]	EEG	Distraction	No	No	No	No	No
[51]	EEG	Distraction	No	No	No	No	No

The BSN column refers to the body sensor signals collected and processed to infer the driver’s emotional or intoxication states. It must be noted that not in all the cases refer to body signals, some projects realise indirect measurements of the driver performance via external instrumentation, for instance the car facilities such as: steering speed, car velocity, braking behavior, *etc.*, or video cameras that allow determine the driver postures.

The Monitoring state column refers to the driver emotional or intoxication state measurements such as: distraction drowsiness, drunkenness, *etc*. It should be noted that because in some projects the car driver state inference is realised on the base of external instrumentation the column holds those variables, for instance, the car kinematics such as the steering speed, velocity speed, braking performance, acceleration, or via the use of cameras installed inside the car cockpit driver that allow determine the driver postures, *etc.*

The Monitoring station column indicates whether there is a project element where the sensor signals are collected, processed and analysed in-situ by the prototype or the data analysis is left for posterior study in a laboratory, for instance.

The OBU column considers whether the project features mechanisms that enable to issue alarms to the VANET, or the authorities and emergency services of potential accidents in course.

The Emergency services column shows whether the platform implements any kind software that enables the interaction with the car facilities (worn, radio, interior lights, engine, *etc.*); in order to make the driver reacts; the VANET, or the pedestrians to prevent potential fatal accidents in course.

The Communication interfaces column considers whether the project incorporates software interfaces and the technologies employed; Bluetooth, Wi-Fi, ZigBee, GPRS or others; to enable communication with the car, external networks, facilities or pedestrians.

The VANET column shows whether the project considers shaping the cars and road or street facilities network up. As it can be observed from Table 2, it is still hard to find projects that merge a global interaction among different sort of networks such as: BSN, VANET, GPRS and the Internet.

## 7. Experimentation

At present, the implementation of the proposed architecture considers the interactions of only one BSN sensor node, the monitoring station, and an Internet server that enable communication links with other networks, for instance the emergency services and the road or street facilities. On the one hand, the sensor node collects data signals and periodically transmits the data to the station. The station is a communication gateway that receives the sensor data, processes it and on the base of the car driver attention critical level issues alarms to the server. On the other hand, the server broadcasts the alarms to the VANET, and pedestrians located nearby the position of the warning station. It follows in the next sections more detailed explanations of the architecture implementations, experimental tests and results.

### 7.1. Monitoring Station Implementation

We have developed an Android real-time attention low level detection application that runs in a next-generation smartphone. The application features mechanisms that allow: to measure the degree of attention of a driver on the base of her/his EEG signals, establish wireless communication links via various standard wireless means, GPRS, Bluetooth and WiFi, and issue alarms of critical low driver attention levels.

In the driving process, the EEG device periodically gathers raw readings of the driver brain waves and sends the readings via a Bluetooth wireless link to the Android application. Once the smartphone has received the EEG data, it processes the data to extract meaningful features; these features then serve as input models that analyse the driver attention levels, then an alert system is triggered if the results indicate that the driver attention is low.

The application works on the base of the attention values that are ranged within a scale of 0 to 100 units. Alarms are issued by the application whether within an adaptable time window, the driver attention value gets critically low levels for determined time durations. Currently, the application performs evaluations over three attention level intervals, (i) values within 40–100 units indicate the driver is fully focused; (ii) values within 20–40 units mean low attention, and iii) critical low attention levels for values less than 10 units.

A client-server communication scheme is used to receive the alarms issued by the monitor and to broadcast message to the VANET, the road or street facilities, and pedestrians. Two sort of alarms are triggered by the application, warning messages and emergency messages. Warning messages are issued when the driver attention levels are within the 20–40 units. Emergency messages are transmitted when the driver attention levels are under the 10 units.

**Figure 5:**
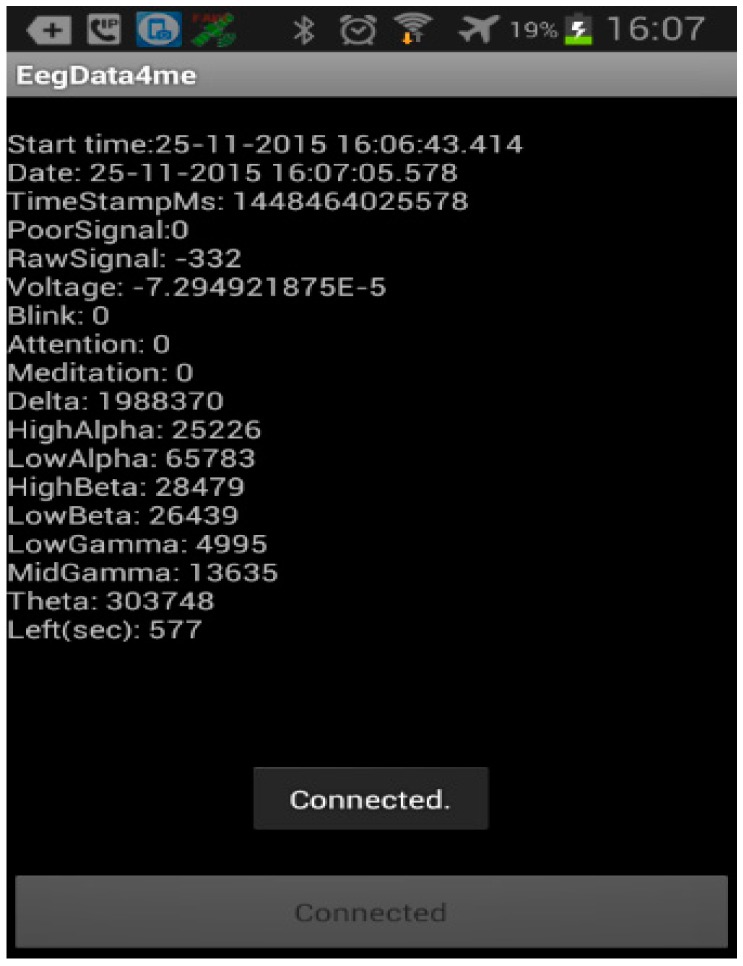
Android application to collect the EEG data.

Whilst warning messages are addressed to the vehicles and pedestrians that are nearby the driver vehicle low levels of attention. Emergency messages are broadcasted to the emergency services, the nearest vehicles (those within a 1 km distance) and pedestrians. Eventually, it is considered to make actions over the vehicle controls such as slowing the car velocity, increasing the car radio volume, switching on the turn and stop lamps, and switch the engine off, for instance. In Figure 5 is shown a snapshot of the data collection interface of the developed Android application.

### 7.2. Instrumentation Device

**Figure 6:**
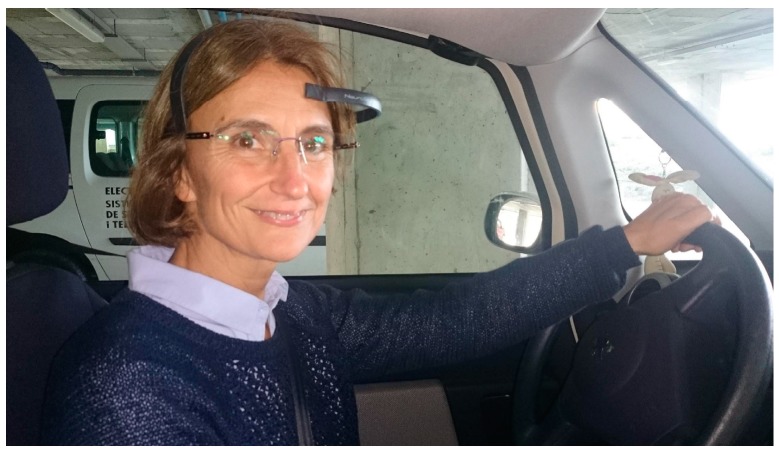
Participant with the EEG acquisition device.

In the experimentation process, the Neurosky Mindwave mobile, that is an unobtrusive low-cost (€136) sensor device for EEG data acquisition is employed, see Figure 6. The device consists of a headband with three sensors, the reference and ground electrodes that are clipped onto the earlobe, whilst the EEG recorder electrode is positioned on the forehead. The sensors require no gel or saline for recording, and no expertise is required for set up. The sensor provides raw EEG signal, alpha, beta, gamma delta and theta, the EEG power spectrum, and the measurements for ‘attention’, ‘meditation’ and ‘eye blink’. Neurosky employs Bluetooth wireless communication which features: a RF data rate of 250 Kbits/s, 10 m RF range and UART baud rate of 57,600 Baud. Attention, meditation, and blink measurements are computed by Neurosky proprietary algorithms with a calculation rate of 1 Hz, and the measurement values range within the scale of 0 to 100 units.

### 7.3. Participants

A total of three healthy women drivers aged between 35 and 50 years old with more than 15 years of experience driving participated in the test. The participants, *P1*, *P2*, and *P3*, were asked to get a full night of sleep time in the lead up to their study appointments. Participants were also requested to avoid drinking alcohol at least 24 h before their test, and abstain from caffeine on the day before of the study. Informed consent was obtained from all participants.

### 7.4. Procedure

**Figure 7:**
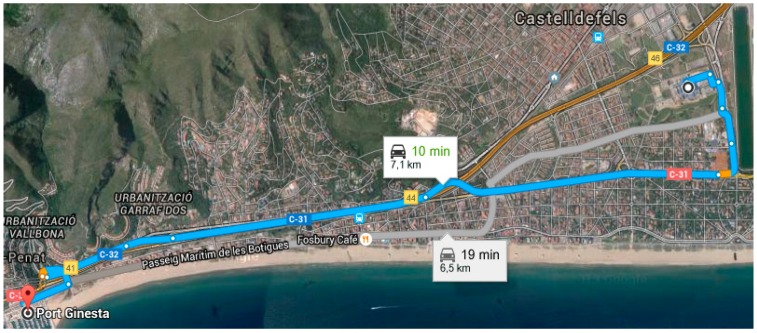
Route of the experiment.

The experiment was developed within three consecutive days starting at 9:30 a.m. Each participant drove during 10 min in her own vehicle. All participants followed the same route from the BarcelonaTech parking to the Port Ginesta of Castelldefels, Spain. See Figure 7. The speed limit that applies to the road-related area was 40 Km/h and the traffic density was very low.

### 7.5. Dataset

**Figure 8:**
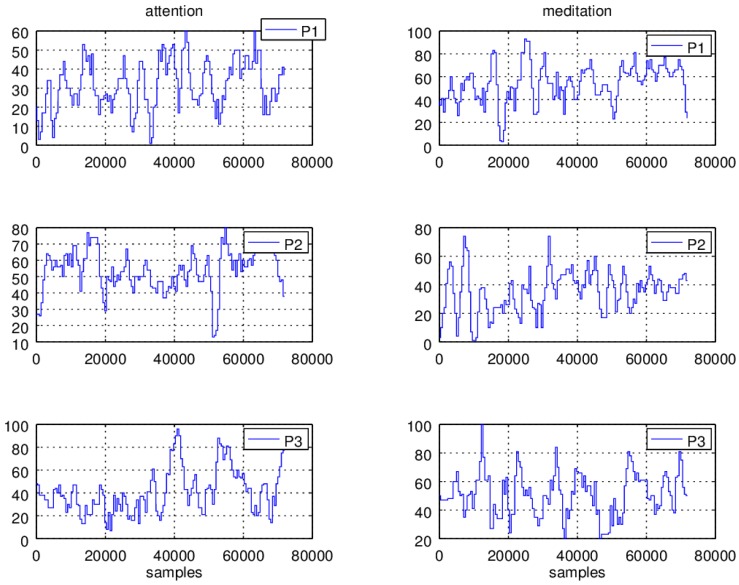
Attention and meditation readings of three individuals, *P1*, *P2*, and *P3* car drivers.

Ten minutes of attention and meditation signals at a sampling rate of 512 samples per second were registered. In Figure 8, two minute readings of the signals of the three car drivers *P1*, *P2* and *P3*, are shown.

In the left column the attention signals are graphed and in the right column the readings of the meditation signals are plotted. In the ordinate, the magnitude of the signals, attention and meditation, are displayed and in the abscissa the sample numbers are shown. In the case of the attention signals, it can be observed that the readings of the *P1* individual are distributed along a value range of 0–60 units, the *P2* readings fall within the range of 0–80 units and the *P3* reading values are allocated within the range of 0–100 units. In the case of meditation values, *P1* presents readings are distributed along 0–90 units, *P2* readings fall within the range of 0–80 units, and *P3* has readings falling within the range of 0–80 units.

From Figure 8, it must be observed that the values apparently do not follow a distinguishable pattern. This is due to the large changes in the signal magnitude and frequency, so the values do not present a predictable behavior and in this manner it is not possible to determine the drivers’ state conditions. Therefore a number of signal processing tasks should be performed before the application can make any decision.

#### Signal Processing

**Figure 9:**
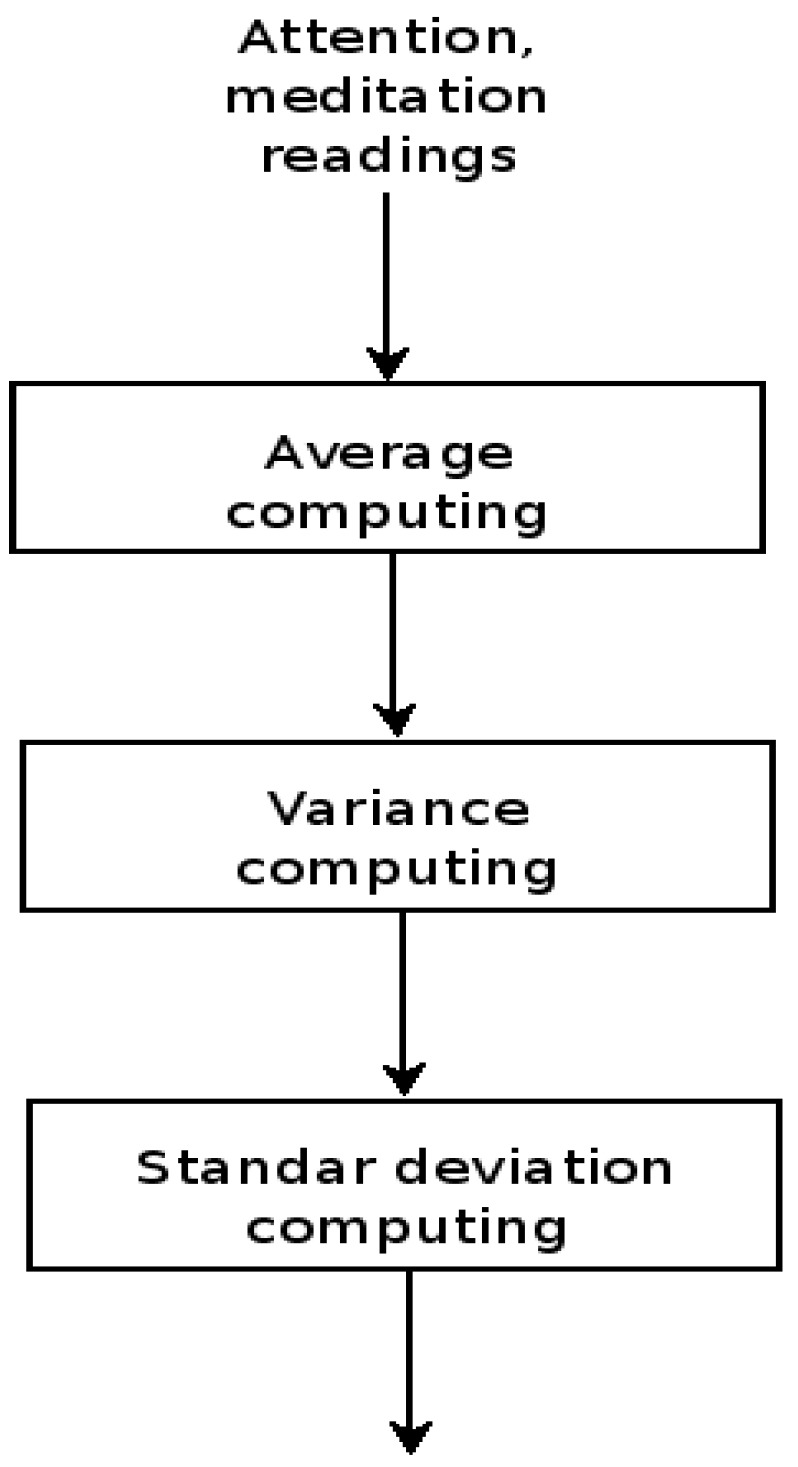
Application signal processing module, adaptive low pass filter, variance and standard deviation computations.

*Signal average*. The values of the readings, attention and meditation, are passed through a point to point averaging module. This procedure has the purpose of cutting high frequency changes out from the signals. This process implies the implementation of low pass filters, more precisely adaptive low pass filters. The outcome is a signal which presents less abrupt changes in the signal amplitude. The average output is passed to the signal variance module, see Figure 9.

*Signal variance*. In the same manner, a software module computes a point to point variance. The purpose of this step leads to enhance the differences between the sensor signal and the average data. In this manner, the variance amplifies little changes in the differences whilst large difference values are kept almost without change. The variance output is passed to the standard deviation module, see Figure 9.

*Signal standard deviation*. In this step a software module calculates a point to point standard deviation. The purpose of this operation is to provide a measurement of the data dispersion.

Figure 9 shows the calculation sequence followed by the application signal processing layer that operates over the signals, attention and meditation of each one of the individuals *P1*, *P2* and *P3*.

**Figure 10:**
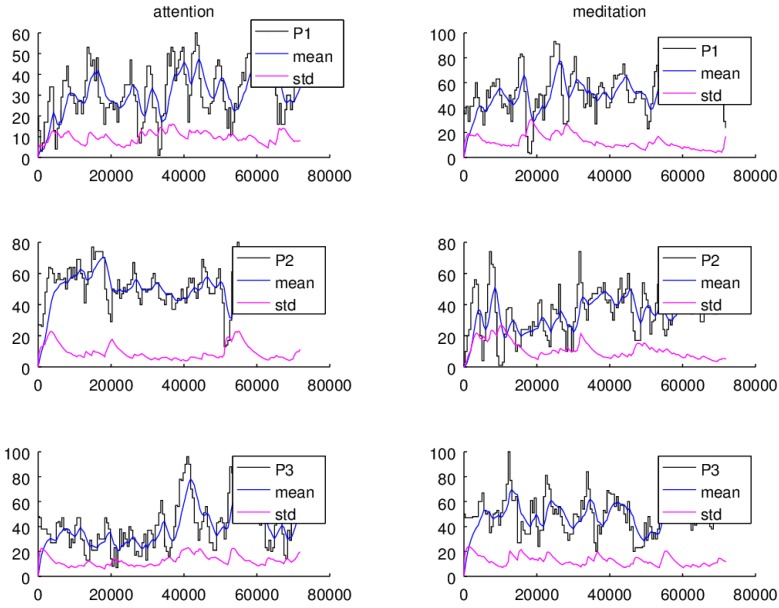
Statistic, average and standard deviation, computations of the attention and meditation signals of the individuals *P1*, *P2*, and *P3*.

The attention and meditation signals, and the average and standard deviation computations for the *P1*, *P2*, and *P3* individuals are plotted in Figure 10. In the ordinate, the magnitude of the signals, attention, meditation, average, and standard deviation, are displayed and the abscissa shows the sample numbers. It should be noticed that the average, and standard deviation adaptive algorithms take around 1536 samples (three seconds) to settle down to the actual signal average and standard deviation values. In both cases, it is observed that the average estimations give a more stable reference with respect the changes observed in the amplitude and frequency of the input signal, attention and meditation readings. In the same manner, the standard deviation of the signals peacefully follows the average computation changes.

Additionally, in both cases it is observed that within some intervals the standard deviation preserves a uniform distance with respect the average values. For instance, it is noticed that individual *P2* can focus (average and standard deviation differences larger than 40 units) for longer time periods (samples 1536 to 50,000) than *P1* and *P3*. Also, it can be observed that in the smallest attention levels the distance between the average and the standard deviation becomes quite closer. On the other hand, the graphs show that *P1* and *P3* are able to keep calm for longer periods of time compared with respect to *P2*, although *P3* presents the lower focusing levels.

Interestingly, running a comparison between the attention and meditation values it is noticed that having high levels of attention does not necessarily imply having higher levels of meditation or peacefulness. In fact, being more focused can be a cause of high stress levels, as it can be seen for individual *P2* whose attention average and standard deviation difference values are the highest, while the meditation average and standard deviation difference values are lower with respect to the individuals *P1* and *P3*, see Figure 10.

### 7.6. Low Level Attention Driving State Detection

In this manner the distance between average and standard deviation values can be employed to have in a way a measurement of the attention level. Furthermore, the observed distance can be combined with other measurements: such as the data collection timestamps, the car speed, orientation, steering speed, *etc.* that can be a measurement of the driver performance.

In the particular case of the developed application two variables are under consideration to detect a possible impaired driving performance: (1) the difference between the attention average and standard deviation values and (2) the elapsed time the driver performs with low attention average and standard deviation values. In this manner, alarms are issued given the condition, large time periods with low attention differences. To do this, on the one hand, the attention difference measurements are grouped within three ranges: (I) the values that fall between 10 and 20 units are considered low; (II) the difference values that are within the 20 and 60 units mean elevated cognitive load and (III) the difference values below 10 units are interpreted as lowered levels. On the other hand, assuming the maximum driving velocity of 40 km/h, approximately the time required to advance a distance of 10 m is close to one second.

## 8. Performance of the Architecture

The performance of the integrated architecture is evaluated in terms of alarm triggering, the BSN bandwidth estimation and the Monitoring Station bandwidth estimation.

### 8.1. Alarm Triggering

The architecture has been tested with the EEG signals of the *P1* driver under two car driving cases: in phase 1 the drivers are asked to just guide the car without any secondary task and in phase 2 the drivers are asked to drive while performing secondary tasks. In both field studies, participants drove while EEG data were recorded.

For phase 2, in addition to the primary driving task, participants had to complete two secondary cognitive demand tasks that increase the workload. Within the first three minutes, the drivers, via the hands-free set, must pick up and answer a phone call that lasts one minute. Then a rest period of 2 min is introduced. The second procedure consists of presenting a digit span test, it means, an auditory presentation of the single digits 0–9, one at a time in a randomly ordered sequence. As each new item was presented, participants were required to say out loud the digit two items back in the current sequence. This secondary task requires auditory perception and cognitive processing involving working memory [76]. The test considers a set of 30 number series of three digits, between each series there is a pause period of two seconds. Finally, the rest of the time the driver is left guiding with no load until she/he arrives the destiny.

**Figure 11:**
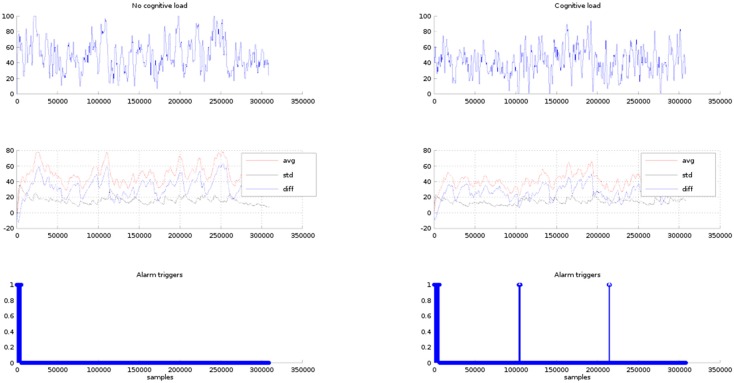
Row 1, *P1* EEG signals for the no cognitive load and cognitive load. Row 2, average, standard deviation and difference of the EEG signal values. Row 3 alarm triggers for difference values of less than 10 units.

In Figure 11, row one, the *P1* EEG signals are plotted for the conditions of phases 1 and 2. In row two, the statistical EEG values (average and standard deviation) and the difference between the average and the standard deviation are graphed. In row three the alarms triggered by the triggering module are shown. The triggering limit is set to a difference value of 10 units. In both cases, phases 1 and 2, within the first 47 s the triggering module issues several alarms. It is because this is the time required by the adaptive average and standard deviation algorithms to adjust their computations to a settled estimation value, therefore these alarms are discarded from the test results. In case of phase 1, after the settling time period has elapsed, as expected there are no alarms triggered along all the resting time of the test. In case of phase 2, within the time period of the phone call, there are no alarms issued. Then, two alarm sets are triggered along the rest of the test time period. The first alarms are centered around the 100,000th sample (6.53 min) and around the 230,000th sample (15.033 min). The alarm at minute 6.53 corresponds to the digit span test and the alarm in the 13.033 min is out of the car guiding period, therefore this may be discarded.

**Figure 12:**
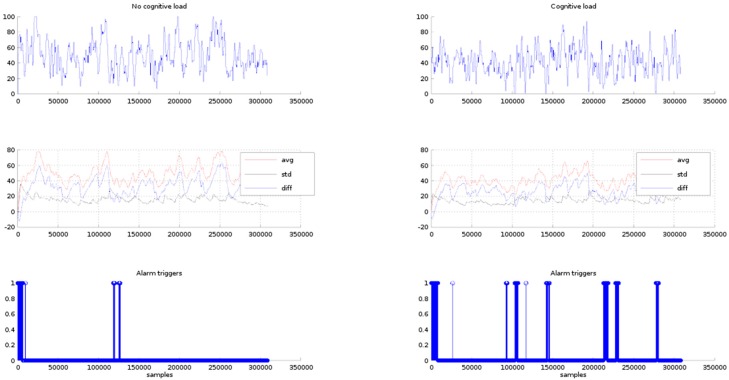
Row 1, *P1* EEG signals for the no cognitive load and cognitive load. Row 2, average, standard deviation and difference of the EEG signal values. Row 3, alarm triggers for difference values less than 15 units.

It must be observed that the triggering module sensibility is controlled by the triggering limit. In fact, if the triggering limit is raised the number of triggers is increased. For instance, in Figure 12, the triggers obtained for the setting of the triggering limit to a value of 15 units are shown in row 3. In the case of phase 1, the triggering module issues alarms within the 100,000th to 150,000th samples interval. These triggers are false positives because the expected result is no alarms under the condition of no cognitive load. In the case of phase 2, there are six points where alarms are triggered, the first set is issued between the 13,000th and 15,000th sample period (50 s to 60 s). The second group is centred at the 100,000th sample (6.53 min). The third group lays around the 150,000th sample (minute 15.033) that is out of the test time period, and the other alarm groups are also outside the test time period, therefore as previously has been mentioned these alarms may be discarded.

**Figure 13:**
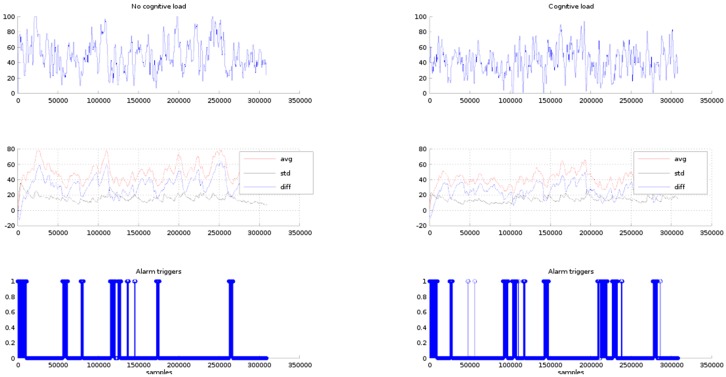
Row 1, *P1* EEG signals for the no cognitive load and cognitive load. Row 2, average, standard deviation and difference of the EEG signal values. Row 3, alarm triggers for difference values less than 20 units.

In Figure 13, row 3, the issued alarms for a triggering limit of 20 units are plotted. In both cases, phase 1 and phase 2, the number of triggers has been incremented, appearing alarms in the places where the difference between the average and standard deviation are less than 20 units. In this case, the alarms appearing in the phase one can all be considered as false positives. In the phase 2, it is expected the triggers appear in the places where the phone call answering and the digit span test take place.

From the previous discussion it can be seen that a tuning process should be run in order to find out the triggering limit that best points the critical degree out of the driver attention.

### 8.2. BSN Bandwidth Estimation

The BSN is made up of one single hop transmission star topology network, with at most seven sensor slaves, and one master played by the monitoring station. It is assumed a 2.4 GHz radio transmission link with data rates within the range of 1 Mbit/s to 3 Mbit/s, such as the featured by the Bluetooth low energy or Zigbee IEEE 802.15.4 RF radio transceivers. It should be observed that with the use of the Bluetooth technology there is no possibility of data packet collisions. In fact, sensors data requests are synchronised by the master in a round-robin fashion. Differently, Zigbee would manage a larger number of slaves rather requiring the implementation of a contention access media protocol.

The prototype architecture considers the monitoring of two physiology EEG signals: drowsy and attention. The EEG sensor requires one second to compute one drowsy and one attention level estimations, then the sensor transmits within the Bluetooth wireless channel a data packet. The data packet structure of the EEG sensor consists of three sections: (i) a 3 bytes header; (ii) a 0–169 bytes payload and (iii) a 1 byte checksum. In the worst of the cases the data payload length of the EEG Bluetooth packet is 173 bytes. Each EEG data inserted in the packet payload is identified by a code number, a data type, and the sensor reading value. The code number is one byte and labels the physiology state, blink or attention, the data type is one byte and shows the variable length, and the value field that is the sensor reading value, generally a two bytes integer value. In our case the data packet payload starts with the blink code, the data type, and the two bytes of the sensor value. Secondly, the attention code, the data type and the two bytes integer sensor value. In this manner, the total data payload packet is eight bytes, two bytes for the command codes, two bytes for the value types, and four bytes for the two state values. The data transmissions among the slaves and the monitoring station are limited to the the RF data rate that is 32 Kbytes per second. Therefore, assuming a full packet payload, the time required to transmit an EEG data packet of payload length is 3.27 ms.

From the discussion in Section 2.4, the respiration monitoring can serve to detect drunkenness and emotional disorders, therefore the design of the prototype considers respiration sensors. The sensor data rate is 800 bps (100 Bps) and the frequency to estimate one state value is around the 0.1–10 Hz (10 s–100 ms). Then, assuming a Bluetooth transmission link, a data packet of length 173, the time required to transmit one sensor data at the maximum frequency, 100 ms, is 103.27 ms.

### 8.3. Monitoring Station Bandwidth Estimation

After receiving the sensor signals, EEG and respiration. Separately, the monitoring station computes the impaired driver degree for each signal. Whether is found in any or both signals critical levels alarms are triggered via an event based detection algorithm. In this case events are abnormalities in the course that potentially would become an incident. In principle, events rarely happen therefore the most of the time the wireless channel links between the monitoring station, the services server, GPRS or WiFi, and the VANET, WiFi, are available.

## 9. Research Challenges

Next, the challenges concerning the integration of BSNs and VANETs are introduced. Information security and privacy preservation are essential challenges. It must be strictly guaranteed that sensitive and private driver-related data is only accessed by authorized users. For this reason, ordinary notification messages should warn about an improper driver’s state but not specify the state or incorporate related body sensor data. In addition, the emergency services should also be notified if driver’s assistance is required and body sensor data should be forwarded. It is essential to ensure the secure delivery of patient-related data, since the inability to securely store and transfer authentic data can prevent the driver from being treated effectively.

However, the integration of a high-level security mechanism in low-power and resource-constrained body sensors increases the computational, communication and management costs [77]. Therefore, simple and effective biometrics-based methods that use the intrinsic human body characteristics for user authentication and secure key cipher transmission should be designed to protect the privacy of drivers. Data should be protected against modifications of malicious vehicles [78] (such as replay attack) and against node failures.

Furthermore, ensuring comfort during driving while preserving security is essential. Safety belts are sometimes considered uncomfortable, since they restrict movement but they prevent deaths in many traffic accidents. The same would apply to BSNs. In addition, as technology evolves, a limited number of highly sensitive, miniaturized sensors will be worn more comfortably and will continuously measure the vital signs and brain activity without disrupting the driver.

Another key challenge is the provision of Quality of Service (QoS) in this architecture. In BSNs loss-sensitive and delay-sensitive applications require efficient error detection strategies to guarantee a high level of QoS. The Bit Error Rate (BER) can be reduced applying error correction and interference-avoidance methods at the MAC and physical layers. End-to-end delay and jitter are important parameters to ensure a fast delivery of high-priority traffic. The link quality conditions are dramatically affected by the spatial and temporal variability of the vehicular channel. If safety-related notification messages are delayed, lost or corrupted, the reliability of the network would be seriously damaged and in a worst case scenario the non-detection of a traffic driving impairment could be fatal. It is required to develop adaptive QoS routing schemes that can quickly redirect traffic when the established routes are no longer available. Important QoS metrics such as the packet delivery ratio and connection duration require in-depth research and improvement.

Furthermore, position-based information dissemination should be accurate and reliable. Since the BSN-VANET integration is strongly dependent on the geographic location of the vehicle that sends notification messages, positional information is crucial. Wrong position information due to malfunction or intentionally falsified by attackers degrades the performance of the system and should be quickly detected and corrected.

The accuracy of the impaired driving detection systems is also challenging. Detecting emotional state disorders is especially difficult, since there is no agreement on how emotions can be measured and classified.

The impact of false positives in real life scenarios should also be analysed. In this sense, body sensor networks technologies have been incrementally improved and tuned to the point where claims of near-perfect accuracy are not unheard of, despite relatively high recording noise levels and high complexity of the collected signals [79]. So there is an increasing need to compare and evaluate the performance of different behavior state detection methods using BSNs. In future work, we will explore the relationship between classification accuracy and driver performance that we think may reveal interesting correlations.

Some legal and ethical issues arise from the BSN-VANET integration. Regulations about the use of BSNs while driving should be defined. Its use could be mandatory for every driver or just restricted to the control of drivers with prior driving while impaired convictions. In this last case, BSNs could be effectively used to prevent accidents, since statistics demonstrate that drivers with a blood alcohol concentration of 0.08% or higher involved in fatal crashes were four times more likely to have a prior driving while impaired conviction than were drivers with no alcohol (8% and 2%, respectively) [80]. The use of BSNs by drivers is also a controversial issue from a social and ethical viewpoint. Many people could be concerned about illicit access or accidental revelation of personal information through the VANET to others, since this occurrence may be undesirable from the driver’s perspective and could violate civil liberties. Since inappropriate use of the drivers’ states could lead to discrimination, the proper degree of protection of these data is essential. Therefore, a regulation that determines the ownership of the data and its legal liabilities should be explicitly defined.

Energy efficiency in BSNs is a challenging issue as well. The battery capacity is limited by the dimension of the nodes. Using human energy harvesting as secondary energy source to supplement batteries sounds promising but arises different challenges such as the amount and availability of the collected energy [81]. In addition, it is possible to improve the energy efficiency and robustness to link failures of BSNs by exploiting the benefits of Random Linear Network Coding (RLNC)-based cooperation and Compressed Sensing (CS)-based signal processing algorithms [82]. RLNC is based on the transmission of linear combinations over a block of original data packets, created by multiplying each packet with a random coefficient drawn by a finite Galois field. RLNC techniques can also be applied to provide reliability. In [83], the authors propose a cloud-assisted MAC protocol that guarantees the delivery of all data packets to the destination by enabling the coordination among the relays of a relay sensor network, that is, a network that enables the transmission of information between a BSN and the medical data center.

## 10. Conclusions

If we focus on the driver behaviours, traffic accidents are likely to happen when drivers make a mistake or manifest improper driving behaviors while they misjudge the risks of driving environments. Such driving behaviours are mainly caused by four factors: drunk driving, drowsy driving, distracted driving and drivers with emotional states disorders.

In this paper an architecture for the integration of BSNs and VANETs to detect driving impairments has been introduced. Its main components and relevant driver’s states have been described. Thanks to this integration it is possible to monitor the driver’s state and take the necessary actions to avoid accidents.

In our BSN-VANET integration proposal four behavior states have been addressed: drowsy, drunk, emotional state disorders and distracted while driving. Future work will detect other human states via the BSN such as abrupt health problems (e.g., heart attack) that produce driving impairment.
